# Efficacy and safety of fecal microbiota transplantation for the treatment of irritable bowel syndrome: an overview of overlapping systematic reviews

**DOI:** 10.3389/fphar.2023.1264779

**Published:** 2023-10-17

**Authors:** Di Zhang, Yan Tang, Xiangyu Bai, Da Li, Mengxue Zhou, Chunmei Yu, Hua Wu

**Affiliations:** ^1^ Affiliated Hospital of Shandong University of Traditional Chinese Medicine, Jinan, China; ^2^ Xuyi County People’s Hospital, Huaian, China; ^3^ Tianjin University of Traditional Chinese Medicine, Tianjin, China; ^4^ Department of Nutrition, Acupuncture and Moxibustion and Massage College & Health Preservation and Rehabilitation College, Nanjing University of Chinese Medicine, Nanjing, China

**Keywords:** irritable bowel syndrome, fecal microbiota transplantation, treatment, evidence, overview

## Abstract

**Aim:** Evidence from overlapping systematic reviews (SRs) and meta-analyses (MAs) has yielded conflicting results on the treatment of irritable bowel syndrome (IBS) with fecal microbiota transplantation (FMT). To thoroughly gather, assess, and synthesize evidence on FMT for IBS, we carried out the present study.

**Methods:** A comprehensive search was conducted in Cochrane Library, Web of Science, PubMed, and Embase from inception to May 2023. Tools for assessing the methodological quality, reporting quality, and confidence in outcomes, including A Measurement Tool to Assess Systematic Reviews 2 (AMSTAR-2), Preferred Reporting Items for Systematic Reviews and Meta-analyses (PRISMA), and the Grading of Recommendations Assessment, Development and Evaluation (GRADE).

**Results:** Seven eligible SRs/MAs were finally included in this overview. By AMSTAR-2, the methodological quality of SRs/MAs included five that were very low quality, one that was low quality, and one that was high quality. According to PRISMA, limitations were associated with items 5 (Method: Protocol and Registration), 8 (Method: Search), and 27 (Funding). In GRADE, a total of 19 outcomes were included in the seven reviews, of which 12 outcomes were low quality and seven outcomes were moderate quality. Imprecision due to small sample size was the primary factor leading to evidence downgrading.

**Conclusion:** We conclude that there is insufficient evidence to determine whether FMT has a more beneficial effect on patient with IBS than placebo treatment. Well-designed, larger trails are needed to provide evidence in this field. In addition, selection of donor, route of administration, dosage, and frequency still need to be determined.

## Introduction

Irritable bowel syndrome (IBS) is a common functional gastrointestinal disorder characterized by altered bowel habits and abdominal pain in the absence of biochemical abnormalities or detectable structural (Mearin et al., 2016). With a worldwide prevalence of approximately 4.1% (Sperber et al., 2021), IBS significantly reduces health-related quality of life (QoL), interferes with work productivity, and places a significant burden on healthcare services (Black and Ford, 2020). The pathogenesis of IBS is s still poorly understood which has posed considerable hurdles in developing effective therapy options (Chey et al., 2015).

Emerging evidence points to altered microbiota composition in IBS patients, implying that the gut microbiota may play a significant role in the etiology of IBS (Jalanka et al., 2015; Tap et al., 2017). As a result, alteration of the composition of the gut microbiota has been advocated as a therapeutic method for IBS (Shaikh et al., 2023). Fecal microbiota transplantation (FMT) is a novel therapy for targeted balancing of intestinal microecological dysregulation that has been recognized as a successful treatment for recurrent *Clostridium difficile* infections (Quraishi et al., 2017). Given the role of the intestinal microbiota in the etiology of IBS, interest in using FMT to treat IBS continues to increase, as does the number of systematic reviews (SRs) and meta-analyses (MAs) on this topic (Ianiro et al., 2019; Xu et al., 2019; Abdelghafar et al., 2022; Samuthpongtorn et al., 2022; Wu et al., 2022; Zhao et al., 2022; Rodrigues et al., 2023).

Publication of a large number of overlapping SRs/MAs on the same topic does not always facilitate the use of evidence. It is a difficult undertaking to identify and understand evidence from the increasing quantity of occasionally redundant, incorrect, or conflicting syntheses (Huang et al., 2021a). Different methodological behaviors and reporting quality can complicate the evidence originating from SRs/MAs (Huang et al., 2020a; Huang et al., 2020b). Therefore, overview of SRs/MAs, a new form of evidence synthesis designed to address this challenge by gathering, evaluating, and synthesizing evidence from multiple SRs/MAs on the same topic (Huang et al., 2020c), is presented. To thoroughly gather, assess, and synthesize evidence on FMT for IBS, we therefore carried out this overview of SRs/MAs.

## Methods

### Registration and protocol

The protocol design of this study followed the Cochrane Handbook (Huang et al., 2020d) and was registered in the PROSPERO database. We reported this overview in accordance with the PRIOR statement (Gates et al., 2022).

### Search strategy

We performed a systematic search of the publications using Web of Science (1900–May 2023), EMBASE (1947–May 2023), PubMed (1946–May 2023), The Cochrane Library (1993–May 2023). Search terms used for FMT were “fecal microbiota transplant” or “faecal microbiota transplant” or “stool transplant” or “fecal transfusion” or “fecal bacteriotherapy.” The results were combined with key words related to IBS. Table 1 shows the search strategy using in PubMed.

**TABLE 1:** Search strategy for the Medicine database.

Query	Search term
#1	Irritable bowel syndrome [Mesh]
#2	Irritable bowel syndrome [Title/Abstract] OR irritable colon syndrome [Title/Abstract] OR irritable colon [Title/Abstract] OR gastrointestinal syndrome [Title/Abstract] OR colon spasm [Title/Abstract] OR allergic colitis [Title/Abstract] OR colon allergy [Title/Abstract] OR IBS [Title/Abstract]
#3	#1 OR #2
#4	Fecal microbiota transplant [Mesh]
#5	Fecal microbiota transplant [Title/Abstract] OR faecal microbiota transplant [Title/Abstract] OR stool transplant [Title/Abstract] OR FMT [Title/Abstract] OR fecal transfusion [Title/Abstract] OR fecal bacteriotherapy [Title/Abstract]
#6	#4 OR #5
#7	Meta-analysis as topic [Mesh]
#8	Meta-analysis [Title/Abstract] OR systematic review [Title/Abstract] OR meta-analyses [Title/Abstract] OR meta analysis [Title/Abstract] OR metaanalysis [Title/Abstract]
#9	#7 OR #8
#10	#3 AND #6 AND #9

### Study selection

The titles and abstracts of all citations found through the literature search were independently examined by two reviewers. The selection criteria were then applied after retrieving studies that might be pertinent and reviewing and include any relevant references. The PICOS criteria of the present overview were as follows: (a) population: patients diagnosed with IBS by Rome III (Drossman, 2006), or Rome IV (Drossman, 2016); (b) intervention: FMT by any route of administration and any dosage; (c) comparison: placebo (autologous transfer, excipients with no microbiota); (d) outcome: reported improvement in global IBS symptoms, IBS symptom severity scale (IBS-SSS), QoL, also the adverse events of the intervention; (e) design: SRs/MAs only included randomized controlled trials (RCTs).

We excluded repeated publications and conference abstracts.

### Data extraction

All of the data were independently extracted into a Microsoft Excel spreadsheet by two reviewers. The publication year, authors, country of origin, sample size, IBS criteria, subtypes, preparation of fecal microbiota and placebo, FMT route and frequency, follow-up, methods for quality assessment, primary outcomes, and main results were all collected from all eligible studies.

## Methodological appraisal

The Assessment of Multiple Systematic Reviews 2 (AMSTAR2) tool (Shea et al., 2017), was applied by two authors independently to evaluate methodological quality. There are 16 items of AMSTAR-2, with seven of them being critical items (2, 4, 7, 9, 11, 13 and 15). The methodological quality of SR was rated on four levels (high, moderate, low and very low) according to the following criteria: (a) very low: more than one critical flaw with or without non-critical weaknesses; (b) low: one critical flaw with or without non-critical weaknesses; (c) moderate: more than one non-critical weakness; (d) high: no or one non-critical weakness.

### Assessment of reporting quality

The Preferred Reporting Items for Systematic Reviews and Meta-Analyses (PRISMA) checklist provides specifications for standardized reporting of SRs/MAs (Page et al., 2021). In the present study, PRISMA checklist was applied by two authors independently to evaluate reporting quality. The PRISMA checklist includes 27 items, each of which is a standardized question with results that can be responded to as “yes,” “partially yes” and “no”.

### Certainty of the evidence

The Grading of Recommendations, Assessment, Development, and Evaluation (GRADE) system (Pollock et al., 2016) was applied by two authors independently to evaluate certainty of the evidence. The GRADE method evaluates limitations, inconsistency of results, indirectness of evidence, imprecision, and reporting bias. The certainty of the evidence can be qualified as very low quality, low quality, moderate quality, and high quality.

## Results

### Results on study selection

**FIGURE 1:**
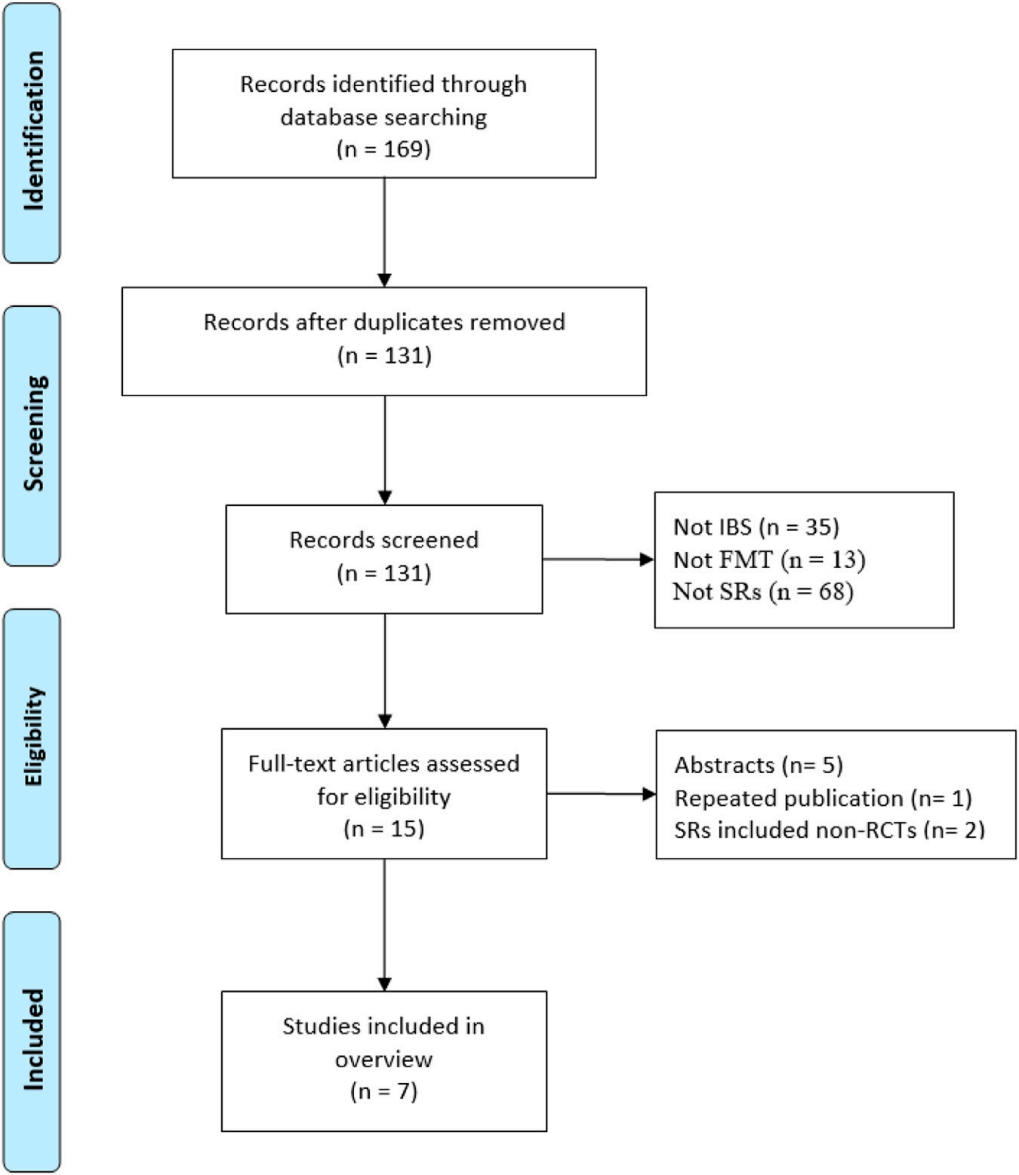
Flow-chart of study selection.

Our initial searches yielded 169 records, and 30 duplicates were removed. Following that, 116 records were excluded based on their titles and abstracts, and 8 records were excluded owing to inconsistency with inclusion criteria. Finally, we included seven SRs in this overview (Ianiro et al., 2019; Xu et al., 2019; Abdelghafar et al., 2022; Samuthpongtorn et al., 2022; Wu et al., 2022; Zhao et al., 2022; Rodrigues et al., 2023). Figure 1 presents the PRISMA flow diagram.

### Characteristics of the included studies

**FIGURE 2:**
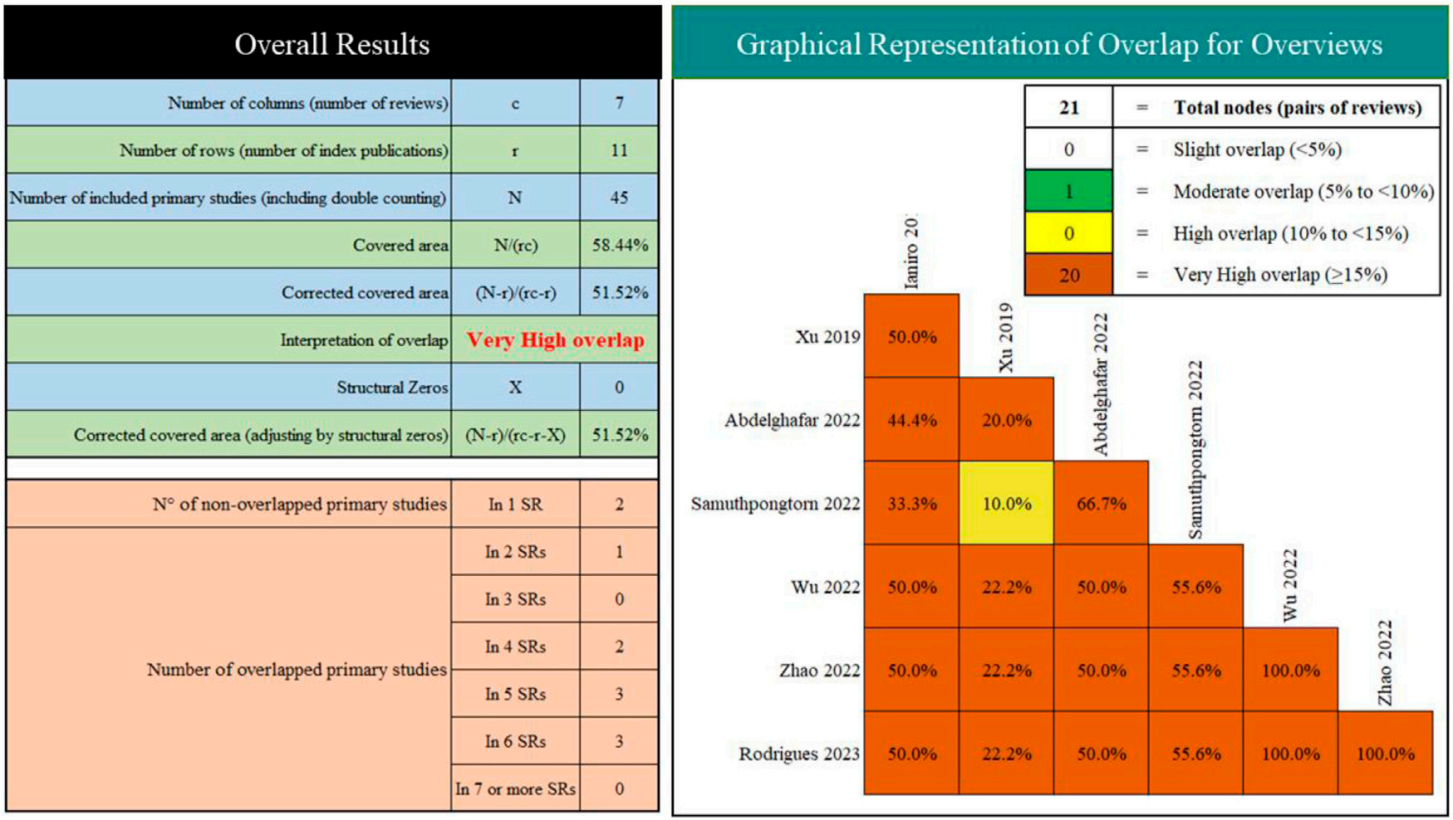
Overlapping of the included reviews.

We included seven SRs/MAs published between 2019 and 2023 (Table 2). Of the included studies, two were conducted in China, one in Italy, one in United States, one in Egypt, one in Thailand, and one in Portugal. Overall, the number of RCTs included varied from four to eight, and the number of participants was from 254 to 505. All studies report quantitative analyses, yet their results are contradictory. Furthermore, we observed very high overlap between the included reviews (Figure 2).

**TABLE 2:** Characteristics of the included reviews.

Studies	Country	Trials (subjects)	Diagnostic criteria	Experimental intervention	Control	Frequency	Follow-up	Outcomes	Conclusion summary
					Intervention	/Duration			
Ianiro, 2019	Italy	5 (267)	Rome III	FMT	Placebo	Once, 25 capsules daily × 3 days	3 months	①, ④	Fresh or frozen donor stool delivered via colonoscopy or nasojejunal tube may be beneficial in IBS. Larger, more rigorously conducted trials of FMT in IBS are needed
Xu, 2019	United States	4 (254)	Rome III	FMT	Placebo	Once, 25 capsules daily × 3 days	6–12 months	①, ④	Current evidence from RCTs does not suggest a benefit of FMT for global IBS symptoms
Abdelghafar, 2022	Egypt	8 (472)	Rome III, Rome IV	FMT	Placebo	Once, 25 capsules daily × 3 days	6–18 months	①, ②, ③, ④	FMT is not an effective treatment to relieve all the symptoms of IBS. Even in the groups that showed relatively significant improvement after FMT, the effect was proven to wear off over time and the re‐administration carries a low success rate
Samuthpongtorn, 2022	Thailand	7 (505)	Rome III, Rome IV	FMT	Placebo	Once, 25 capsules daily × 3 days	3–6 months	②, ③, ④	This meta-analysis of RCTs showed that FMT had significant advantages in terms of clinical and endoscopic remission in patients with mild to moderate active UC.
Wu, 2022	China	7 (472)	Rome III, Rome IV	FMT	Placebo	Once, 25 capsules daily × 3 days	6–12 months	1, ③, ④	IBS patients may benefit from FMT when administered via colonoscopy or gastroscope. FMT may improve the quality of life of IBS. The long-term use of FMT in IBS warrants further investigation
Zhao, 2022	China	7 (489)	Rome III, Rome IV	FMT	Placebo	Once, multiple (lasting 3 days)	3–12 months	1, ②, ③	The current evidence from RCTs with all routes of FMT does not show significant global improvement in patients with IBS. However, FMT operated by invasive routes significantly improved global IBS symptoms
Rodrigues, 2023	Portugal	7 (489)	Rome III, Rome IV	FMT	Placebo	Once, 25 capsules daily × 3 days	4–12 months	①, ④	This meta-analysis revealed a set of critical steps that could affect the efficacy of FMT as clinical procedure to treat IBS, nevertheless more RCTs are needed

①:Reported improvement in global IBS, symptoms; ②: IBS, symptom severity scale; ③:quality of life; ④: adverse events.

## Results of methodological quality assessment

Of the included studies, one was rated high methodological quality, one was very low methodological quality, and five were very low methodological quality. The absence of an explicit protocol, a detailed search strategy, a list of excluded studies, and information on funding sources were all common critical flaws. More details of the results of methodological quality assessment are presented in Table 3.

**TABLE 3:** Quality assessment of the included reviews by the AMSTAR-2 tool.

Author, year	AMSTAR-2																Quality
	Q1	Q2	Q3	Q4	Q5	Q6	Q7	Q8	Q9	Q10	Q11	Q12	Q13	Q14	Q15	Q16	
Ianiro, 2019	Y	N	Y	PY	Y	Y	N	Y	Y	N	Y	Y	Y	Y	Y	PY	Very low
Xu, 2019	Y	N	Y	PY	Y	Y	N	Y	Y	Y	Y	Y	Y	Y	Y	Y	Very low
Abdelghafar, 2022	Y	N	Y	Y	Y	Y	N	Y	Y	N	Y	Y	Y	Y	Y	PY	Very low
Samuthpongtorn, 2022	Y	Y	Y	Y	Y	Y	Y	Y	Y	Y	Y	Y	Y	Y	Y	Y	High
Wu, 2022	Y	N	Y	PY	Y	Y	N	Y	Y	Y	Y	Y	Y	Y	Y	Y	Very low
Zhao, 2022	Y	N	Y	PY	Y	Y	N	Y	Y	Y	Y	Y	Y	Y	Y	Y	Very low
Rodrigues, 2023	Y	Y	Y	Y	Y	Y	N	Y	Y	Y	Y	Y	Y	Y	Y	Y	Low

Y: yes; PY: partial yes; N: no.

## Results of the reporting quality assessment

According to PRISMA, 24 out of 27 items were reported in 100% completeness. However, there were limitations associated with items 5 (Method: Protocol and Registration), 8 (Method: Search), and 27 (Funding). Overall, the included Sas/MAs contained relatively complete reporting quality. More details of the results of reporting quality assessment are presented in Table 4.

**TABLE 4:** Results of the reporting quality.

Section/topic	Items	Ianiro, 2019	Xu, 2019	Abdelghafar, 2022	Samuthpongtorn, 2022	Wu, 2022	Zhao, 2022	Rodrigues, 2023	Compliance (%)
Title	Q1. Title	Y	Y	Y	Y	Y	Y	Y	100
Abstract	Q2. Structured summary	Y	Y	Y	Y	Y	Y	Y	100
Introduction	Q3. Rationale	Y	Y	Y	Y	Y	Y	Y	100
	Q4. Objectives	Y	Y	Y	Y	Y	Y	Y	100
Methods	Q5. Protocol and registration	N	N	N	Y	N	N	Y	28.6
	Q6. Eligibility criteria	Y	Y	Y	Y	Y	Y	Y	100
	Q7. Information sources	Y	Y	Y	Y	Y	Y	Y	100
	Q8. Search	PY	PY	Y	Y	PY	PY	Y	42.9
	Q9. Study selection	Y	Y	Y	Y	Y	Y	Y	100
	Q10. Data collection process	Y	Y	Y	Y	Y	Y	Y	100
	Q11. Data items	Y	Y	Y	Y	Y	Y	Y	100
	Q12. Risk of bias in individual studies	Y	Y	Y	Y	Y	Y	Y	100
	Q13. Summary measures	Y	Y	Y	Y	Y	Y	Y	100
	Q14. Synthesis of results	Y	Y	Y	Y	Y	Y	Y	100
	Q15. Risk of bias across studies	Y	Y	Y	Y	Y	Y	Y	100
	Q16. Additional analyses	Y	Y	Y	Y	Y	Y	Y	100
Results	Q17. Study selection	Y	Y	Y	Y	Y	Y	Y	100
	Q18. Study characteristics	Y	Y	Y	Y	Y	Y	Y	100
	Q19. Risk of bias within studies	Y	Y	Y	Y	Y	Y	Y	100
	Q20. Results of individual studies	Y	Y	Y	Y	Y	Y	Y	100
	Q21. Synthesis of results	Y	Y	Y	Y	Y	Y	Y	100
	Q22. Risk of bias across studies	Y	Y	Y	Y	Y	Y	Y	100
	Q23. Additional analysis	Y	Y	Y	Y	Y	Y	Y	100
Discussion	Q24. Summary of evidence	Y	Y	Y	Y	Y	Y	Y	100
	Q25. Limitations	Y	Y	Y	Y	Y	Y	Y	100
	Q26. Conclusions	Y	Y	Y	Y	Y	Y	Y	100
Funding	Q27. Funding	N	Y	N	Y	Y	Y	Y	71.4

Y: yes; PY: partial yes; N: no.

## Results of the evidence quality assessment

The GRADE system was used to evaluate 19 outcomes of the included SRs/MAs. Of these outcomes, seven were of moderate quality, 12 were of low quality, and no outcome was categorized as high quality. Imprecision due to small sample size was the primary factor leading to evidence downgrading, followed by inconsistency. More details of the results of evidence quality assessment are presented in Table 5.

**TABLE 5:** Results of evidence quality.

Review	Outcomes	№ of trails	Certainty assessment					№ of patients		Relative effect (95% CI)	Quality
			Limitations	Inconsistency	Indirectness	Imprecision	Publication bias	Experimental	Control		
Ianiro, 2019	Response rate	5	No	Serious^b^	No	Serious^c^	No	158	109	RR 0.98 [0.58, 1.66]	⊕⊕⊕○○
											Low
	Adverse events	3	No	Serious^b^	No	Serious^c^	No	94	65	RR 0.93 [0.45, 1.92]	⊕⊕⊕○○
											Low
Xu, 2019	Response rate	4	No	Serious^b^	No	Serious^c^	No	152	102	RR 0.93 [0.48, 1.79]	⊕⊕⊕○○
											Low
	Adverse events	2	No	No	No	Serious^c^	No	84	54	RR 0.96 [0.88, 1.04]	⊕⊕⊕⊕○
											Moderate
Abdelghafar, 2022	IBS‐SSS	5	No	Serious^b^	No	Serious^c^	No	234	158	MD -3.04 [-81.65, 75.57]	⊕⊕⊕○○
											Low
	QoL	4	No	No	No	Serious^c^	No	199	123	MD 9.32 [4.08, 14.55]	⊕⊕⊕⊕○
											Moderate
	Response rate	4	No	Serious^b^	No	Serious^c^	No	176	128	RR 1.12 [0.44, 2.83]	⊕⊕⊕○○
											Low
	Adverse events	5	No	No	No	Serious^c^	No	219	140	RR 1.28 [0.78, 2.12]	⊕⊕⊕⊕○
											Moderate
Samuthpongtorn, 2022	IBS‐SSS	5	No	Serious^b^	No	Serious^c^	No	251	231	SMD -0.58 [-1.09, −0.08]	⊕⊕⊕○○
											Low
	QoL	4	No	No	No	Serious^c^	No	194	177	SMD 0.67 [0.43, 0.91]	⊕⊕⊕○○
											Low
	Response rate	7	No	Serious^b^	No	No	No	302	258	RR 0.63 [0.39, 1.00]	⊕⊕⊕⊕○
											Moderate
Wu, 2022	Response rate	7	No	Serious^b^	No	No	No	290	186	RR 0.75 [0.43, 1.31]	⊕⊕⊕⊕○
											Moderate
	QoL	5	No	No	No	Serious^c^	No	206	131	MD 9.39 [3.86, 14.91]	⊕⊕⊕⊕○
											Moderate
	Adverse events	5	No	Serious^b^	No	Serious^c^	No	222	144	RR 1.20 [0.59, 2.47]	⊕⊕⊕○○
											Low
Zhao, 2022	Response rate	7	No	Serious^b^	No	No	No	234	186	RR 1.34 [0.75, 2.41]	⊕⊕⊕○○
											Low
	IBS‐SSS	4	No	Serious^b^	No	Serious^c^	No	121	128	MD 15.58 [-66.74, 97.91]	⊕⊕⊕○○
											Low
	QoL	4	No	Serious^b^	No	Serious^c^	No	143	123	MD 3.41 [-18.24, 25.07]	⊕⊕⊕○○
											Low
Rodrigues, 2023	Response rate	7	No	Serious^b^	No	No	No	298	191	RR 1.35 [0.75, 2.43]	⊕⊕⊕⊕○
											Moderate
	Adverse events	5	No	Serious^b^	No	Serious^c^	No	232	147	RR 0.88 [0.55, 1.41]	⊕⊕⊕○○
											Low

IBS-SSS: IBS, symptom severity scale; QoL: quality of life. a: the experimental design had a large bias in random, distributive findings or was blind; b: the confidence interval overlaps less, the heterogeneity test *P* was very small, and the *I* ^*2*^ was larger; c: the Confidence interval was not narrow enough, or the simple size is too small; d: funnel graph asymmetry, or fewer studies were included and there may have been greater publication bias.

### Efficacy and safety of interventions

The response rate was assessed by all SRs/MAs, and their results consistently indicated no statistical difference between the FMT group and the placebo group (Ianiro et al., 2019; Xu et al., 2019; Abdelghafar et al., 2022; Samuthpongtorn et al., 2022; Wu et al., 2022; Zhao et al., 2022; Rodrigues et al., 2023). As assessed by three SRs/MAs, one review reported that FMT significantly improved IBS-SSS compared to placebo (Samuthpongtorn et al., 2022), while the other two reviews indicated no statistical difference between the two treatments (Abdelghafar et al., 2022; Zhao et al., 2022). As assessed by four SRs/MAs, three reviews reported that FMT significantly improved QoL compared to placebo (Abdelghafar et al., 2022; Samuthpongtorn et al., 2022; Wu et al., 2022), while the other review indicated no statistical difference between the two treatments (Zhao et al., 2022). The rate of adverse events was assessed by five SRs/MAs, and their results consistently indicated no statistical difference between the FMT group and the placebo group (Ianiro et al., 2019; Xu et al., 2019; Abdelghafar et al., 2022; Wu et al., 2022; Rodrigues et al., 2023).

## Discussion

Evidence from high quality sources tends to prioritize SR/MAs (Yang et al., 2019; Pan et al., 2023). However, the evidence is complex and sometimes redundant, misleading, and conflicting, which may be attributed to the different methodological qualities and reporting behavior of SRs/MAs (Huang et al., 2021b; Huang et al., 2022a; Huang et al., 2022b). Under the circumstances, a systematic overview of these SRs/MAs is urgent needed. In addition, an overview can provide current deficiencies to improve and guide future high-quality SRs/MAs.

### No definitive conclusion can be drawn

There is insufficient evidence to judge whether FMT is effective in treating IBS. Although there was a very high overlap of RCTs contained in the included SRs/MAs, the findings of these reviews were inconsistent. The IBS-SSS was evaluated by the three SRs/MAs included, yet their results are contradictory. Similarly, QoL was evaluated by the four SRs/MAs included, and they have conflicting results. Although response rate was reported consistently by all included SRs/MAs, they all suggested that FMT did not have an improving effect on IBS. Furthermore, the differences in the definition of effective rate among the included SRs/MAs limit the reference value of this outcome. Fortunately, the included SRs/MAs consistently reported that FMT is safe for the treatment of IBS, which seems to build confidence for further studies in the future. The results of the methodological quality evaluation suggested that six of the seven included SRs/MAs were rated as low or very low owing to the deficiencies in items 2 (structured), 4 (objectives), and 7 (sources of information). In addition, almost all included studies were not reported items of 5 (Protocol and registration), 8 (Search), and 27 (Funding) in accordance with recommendations of the PRISMA statement. The GRADE evaluation results suggested low to moderate quality of evidence for outcome and that small sample sizes are to blame for the lack of convincing evidence. Therefore, with the unsatisfactory methodological quality, reporting quality, and evidence quality of SRs/MAs, we do not have sufficient confidence that their results are reliable. No definitive conclusion can be drawn, caution is required when recommending FMT as a complementary treatment for IBS.

### Research gaps to be addressed

The findings of this study suggest that there is considerable scope for addressing methodological and reporting quality issues in the process of SRs/MAs. The availability and credibility of evidence from SRs/MAs can be limited by methodological flaws, and underreporting of SRs/MAs may overstate the effectiveness of the intervention or report no adverse effects, and ultimately reduce the value of the research. Common areas for improvement were evident in the included SRs/MAs. With AMSTAR-2, the lack of a pre-designed protocol, the lack of a detailed search strategy available for replication, the lack of a list of excluded publications, and the failure to report funding sources were common deficiencies in the included SRs/MAs that we identified. According to PRISMA results, the included SRs/MAs have obvious reporting deficiencies, particularly regarding the registration of protocol, retrieval process and source of funding. Furthermore, the results of GRADE evaluation on the quality of evidence suggest that imprecision due to small sample size is the main factor leading to evidence degradation. Therefore, there is considerable scope for improvement in SRs/MAs that are strictly implemented in accordance with AMSTAR-2 and PRISMA and RCTs that are rigorously designed and implemented.

### Implications for research and practice

Based on the above identified deficiencies, researchers should conduct SRs/MAs and report fully in accordance with AMSTAR 2 and PRISMA requirements. In particular, researchers should register or publish research protocols in advance to demonstrate the transparent process of SRs/MAs. The specific search strategy used to implement the literature search needs to be provided, not only to help evaluate its scientific validity, but also to ensure its reproducibility. A list of excluded publications should be provided with an explanation, as this is what a rigorous SR must require. In addition, any potential conflict of interest or funding source issues should be declared. Finally, the sample size of the original RCTs on FMT for IBS should be expanded, which is fundamental to improve the quality of the evidence. Meta-regression or subgroup analysis should be performed if there is significant heterogeneity. When significant heterogeneity is observed, meta-regression or subgroup analysis should be conducted to explore the source of heterogeneity. Future new and updated SRs/MAs should focus on improving methodological quality and reporting quality and avoid multiple overlapping reviews.

The effectiveness and safety of FMT for IBS can be affected by many variables. These variables mainly include materials of FMT, route of FMT, and stool dose and frequency of FMT (Körner and Lorentz, 2023). The diversity of protocols for FMT also limits the overall applicability of the evidence for IBS. Stools and capsules are the two most common styles of materials of FMT. Of the included studies, the vast majority used stools FMT. Interestingly, both stools and capsules were found to be effective in patients with IBS (Ianiro et al., 2019; Xu et al., 2019; Abdelghafar et al., 2022; Samuthpongtorn et al., 2022; Wu et al., 2022; Zhao et al., 2022; Rodrigues et al., 2023). Unfortunately, the lack of sufficient data for extraction prevented a comparison examination of these two FMT materials. There are multiple routes of FMT administration, including oral capsules, enemas, endoscopy, and nasojejunal tube (Ianiro et al., 2019; Xu et al., 2019; Abdelghafar et al., 2022; Samuthpongtorn et al., 2022; Wu et al., 2022; Zhao et al., 2022; Rodrigues et al., 2023). The routes of FMT were then associated with the IBS-SSS score after 3 months of treatment, according to the pooled results (Ianiro et al., 2019; Samuthpongtorn et al., 2022; Zhao et al., 2022). While FMT employing endoscopy has been found to be more effective than enemas and nasojejunal tubes, FMT utilizing endoscopes and capsules has been found to be equally effective (Ramai et al., 2021). Single fecal doses for the studies included varied from 30 g to 80 g for the fecal FMT group and from 9.5 g to 50 g for the capsule FMT group. Neither the total stool dose of FMT nor the single stool dosage of FMT were related to the IBS-SSS score, according to the pooled results (Ianiro et al., 2019; Xu et al., 2019; Abdelghafar et al., 2022; Samuthpongtorn et al., 2022; Wu et al., 2022; Zhao et al., 2022; Rodrigues et al., 2023). Additionally, the frequency of FMT varied between the included studies, which may have contributed to the variations in outcomes. Therefore, the establishment of a standardized and unified FMT protocol is more conducive to the promotion and application of FMT in IBS. In addition, follow-up and further exploration to analyze the long-term effects of FMT are also key points that need to be clarified.

The modifiable factor that induces the development of IBS is diet. Gluten-free foods and low fermentable oligosaccharides, disaccharides, monosaccharides and polyols (FODMAP) have been recognized as the two major dietary plans for inducing remission of IBS (Dionne et al., 2018). As short-chain carbohydrates that are poorly absorbed in the intestinal lumen, FODMAPs are highly permeable and therefore tend to induce abdominal pain and bloating (Marsh et al., 2016). In addition, by interacting with the intestinal microbiota, FODMAPs can lead to gas production and aggravate bloating (Staudacher et al., 2012). Patients with IBS have reported a 68% reduction in symptoms and a significant improvement in quality of life after receiving a low FODMAP diet (Staudacher et al., 2012). Patients who consume gluten without celiac disease develop symptoms of irritable bowel syndrome, a phenomenon defined as “non-celiac gluten sensitivity” (Marchioni Beery and Birk, 2015). However, among human leukocyte antigen (HLA)-DQ2/8-positive patients, a gluten-free diet was effective primarily for the diarrhea subtype and dramatically reduced diarrhea symptoms (Vazquez-Roque et al., 2013). Therefore, FMT should also be fully integrated with dietary modification strategies to better improve symptoms in patients with IBS.

### Strengths and limitations

To the best of our knowledge, this is the first overview to evaluate the evidence of FMT for IBS. By systematically collecting, evaluating, and synthesizing the evidence for FMT for IBS, the findings of this study will aid in evidence-based decision making. However, the evaluation of the quality of included reviews is a subjective process and different researchers may have their own opinions on each factor, although our overview was assessed by two independent researchers.

## Conclusion

We conclude that there is insufficient evidence to determine whether FMT has a more beneficial effect on patient with IBS than placebo treatment. Well-designed, larger trails are needed to provide evidence in this field. In addition, selection of donor, route of administration, dosage, and frequency still need to be determined.

## Data Availability

The original contributions presented in the study are included in the article/supplementary material, further inquiries can be directed to the corresponding author.
